# Double invasion: an unprecedented case of metastases from two distinct primary sites coexisting in a single cutaneous lesion^؆^

**DOI:** 10.1016/j.abd.2025.501195

**Published:** 2025-08-18

**Authors:** Matheus Alves Pacheco, Amanda Amaro Pereira, Leonardo Simas Abi Saab, Rúbia Tabata Rigatti, Oscar Cardoso Dimatos, Athos Paulo Santos Martini

**Affiliations:** a Service of Dermatology, Hospital Universitário Professor Polydoro Ernani de São Thiago, Universidade Federal de Santa Catarina, Florianópolis, SC, Brazil; b Department of Pathology, Hospital Universitário Professor Polydoro Ernani de São Thiago, Universidade Federal de Santa Catarina, Florianópolis, SC, Brazil; c Dermatological Surgery Outpatient Clinic, Dermatology Service, Hospital Universitário Professor Polydoro Ernani de São Thiago, Universidade Federal de Santa Catarina, Florianópolis, SC, Brazil; d Bullous Diseases Outpatient Clinic, Dermatology Service, Hospital Universitário Professor Polydoro Ernani de São Thiago, Universidade Federal de Santa Catarina, Florianópolis, SC, Brazil

Dear Editor,

This case reports a unique presentation, in which a single cutaneous tumor was formed by metastases from two independent neoplasms from different sites. The patient was an 80-year-old man with two tumor lesions on the scalp, with a three-month duration (Fig. 1). He had a history of smoking and renal clear cell carcinoma, diagnosed 16 months earlier, treated with radical nephrectomy and retroperitoneal lymphadenectomy with no evidence of lymph node metastasis. He reported significant weight loss but denied other systemic symptoms.

**Fig. 1:**
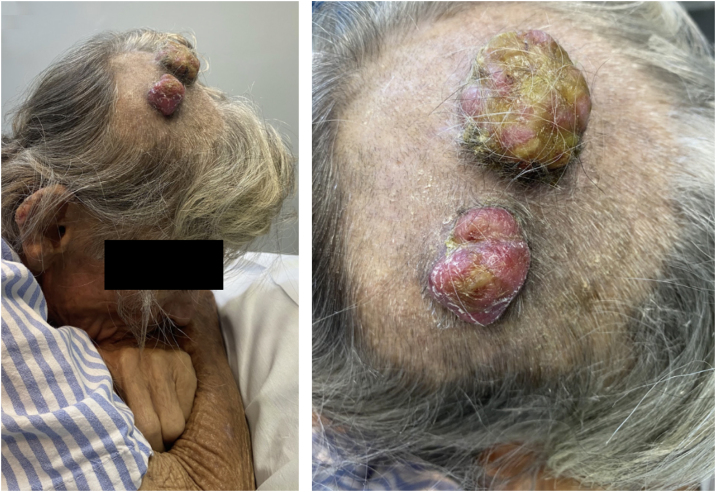
Scalp tumor lesions: Both were well-adhered, firm, friable, and painless. The larger lesion was formed by contiguous metastases of renal cell carcinoma and intestinal adenocarcinoma; the smaller lesion was formed solely by renal cell carcinoma cells.

The lesions were surgically excised *en bloc*, with closure using a full-thickness skin graft. Histopathological examination revealed two dermal lesions with cells with clear cytoplasm, pleomorphic nuclei, prominent vasculature, and geographic necrosis. Immunohistochemistry for CD10 and PAX-8 confirmed metastatic renal clear cell carcinoma (RCC). Beneath the largest lesion, there was a dermal proliferation of atypical cells forming glandular structures, with pleomorphism, mitotic activity, and intraluminal necrosis. Immunohistochemical staining was positive for CK20, CDX2, and SATB2, consistent with metastatic colorectal adenocarcinoma (Fig. 2, Fig. 3).

**Fig. 2:**
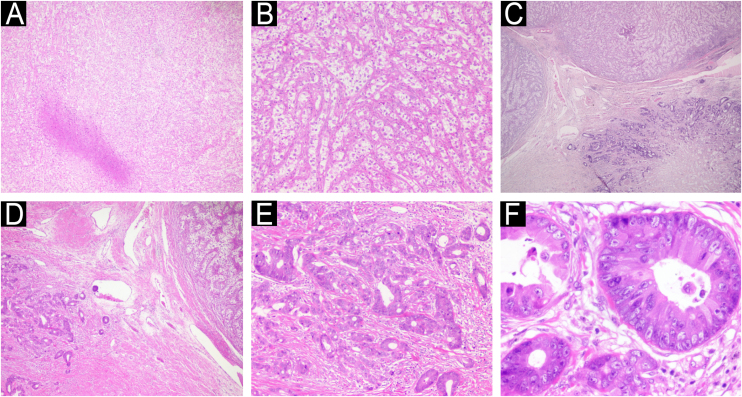
Histopathology of the largest lesion on the scalp. (A) Neoplasm formed by clear cells with an area of geographic necrosis. (B) Clear cells with pleomorphic nuclei permeated by prominent vascularization. (C) Under the clear cell carcinoma, neoplasm with a tubular pattern. (D) Area of angiolymphatic embolization between the two neoplasms. (E‒F) Adenocarcinoma with nuclear pleomorphism and atypical mitotic activity.

**Fig. 3:**
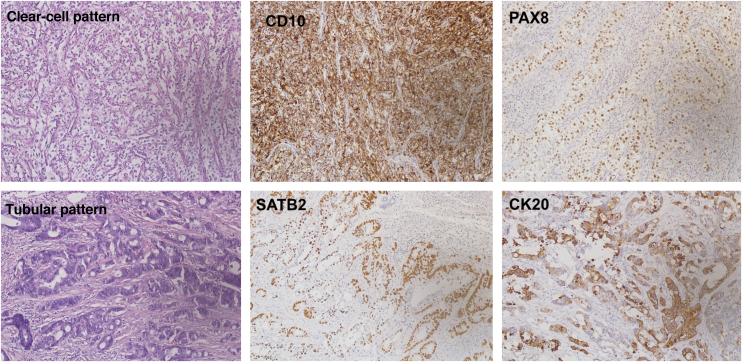
Different immunohistochemical expression profile between the two neoplasms, with positivity for CD10 and PAX8 in the clear cell carcinoma, and for SATB2 and CK20 in the adenocarcinoma.

Colonoscopy revealed an infiltrative lesion in the distal rectum, confirmed by biopsy as colorectal adenocarcinoma. CT staging revealed lung and liver metastases. One month after surgery, despite clear margins, three new metastatic implants appeared at the edge of the graft (Fig. 4). The patient was referred to oncology for palliative treatment.

**Fig. 4:**
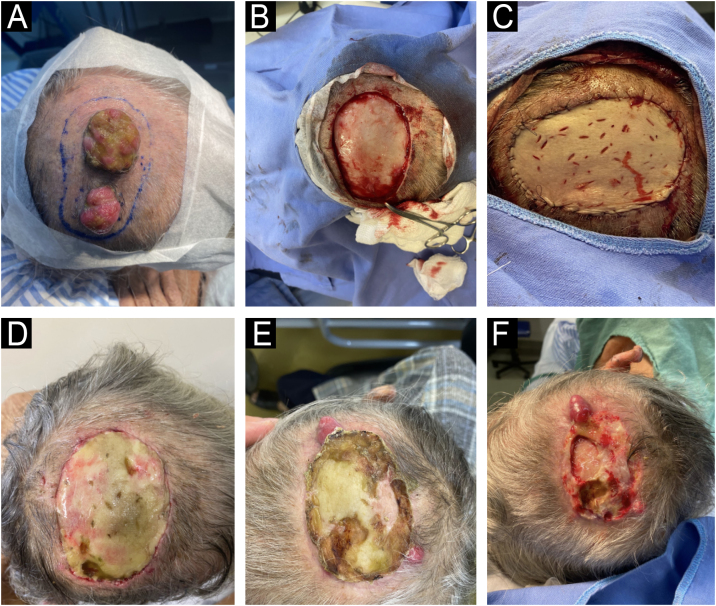
(A) Preoperative planning of excised lesions. (B) Intraoperative view showing *en bloc* resection of lesions. (C) Immediate postoperative result with wound closure using full-thickness skin graft. (D‒F) Late postoperative evolution, showing the emergence of new metastatic implants at the graft edge.

Cutaneous metastases generally appear after the diagnosis of the primary tumor, but in up to one-third of cases, they can be the first manifestation of cancer. In 79% of patients, they are associated with concomitant visceral metastases.^1^ The incidence of cutaneous metastases ranges from 0.7% to 9% of patients with malignancies. The most common tumors vary between the sexes: in men, lung (24%–28.6%), gastrointestinal tract (14.2%–19%), melanoma (13%–18.2%), and head and neck squamous cell carcinoma (9.1%–12%); in women, breast (69%), colorectal (9%), melanoma (5%), and ovarian cancer (4%).^2^

Renal cell carcinoma represents 2%–3% of solid neoplasms in adults, with the skin being the third most common site of metastasis, after lymph nodes and lung (6%–11%).^3^ Clinically, it presents as reddish or purplish, pulsatile nodules located on the extremities, face, scalp, or scars, and may simulate angiomas and pyogenic granulomas.^3^, ^4^ Immunohistochemically, it is positive for PAX8, keratin, EMA, RCC-Ma, CD10, vimentin, and S100, and negative for CK7, CK20, inhibin, Melan-A, calretinin, and TTF-1.^2^, ^5^

Colorectal adenocarcinoma is the gastrointestinal tumor most prone to cutaneous metastases (4% of cases).^6^ They generally arise after the diagnosis of the primary tumor, manifesting as nodules in the abdomen, pelvis, or umbilical region, including the classic Sister Mary Joseph nodule.^5^, ^7^ More rarely, they occur on the face or in scars. Immunohistochemically, they are positive for SATB2, CK20, CDX2, mucin, and CEA, and negative for CK7.^2^, ^8^

The coexistence of metastases from two distinct primary sites in a single skin lesion, as observed in this case, can be explained by metastatic progression models. According to the clonal expansion and rare variant hypotheses, genetic and epigenetic alterations confer selective advantages to tumor cells, enabling angiogenesis, immune evasion, and tissue invasion. These advantages depend on the interaction between tumor cells and tissue microenvironment, mediated by molecules such as integrins, angiogenic growth factors, and cell adhesion proteins.^2^

In this case, the scalp, being a highly vascularized region, may have offered a favorable environment for tumor development. Furthermore, the initial renal cell carcinoma metastasis may have caused local changes, such as vascular stasis, creating a favorable microenvironment that facilitated the subsequent implantation of metastatic colorectal adenocarcinoma cells.

Alternatively, the simultaneous presence of both neoplasms may be the result of a contingency process, in which immunosenescence and microenvironmental alterations favor the independent but concomitant occurrence of these metastases.

This case highlights the importance of a detailed diagnostic evaluation by a dermatopathologist, with a comprehensive examination of the excised lesions to identify distinct cell patterns and guide the immunohistochemical investigation. Future studies are needed to expand our understanding of the mechanisms of interaction between malignant cells and the cutaneous microenvironment, as well as their clinical implications.

## Research data availability

Does not apply.

## Scientific associate editor

Ana Maria Roselino.

## Financial support

This research did not receive any specific financial support from public, private, or non-profit funding agencies.

## Authors’ contributions

Matheus Alves Pacheco: Design and planning of the study; drafting and editing of the manuscript or critical review of important intellectual content.

Amanda Amaro Pereira: Design and planning of the study; drafting and editing of the manuscript or critical review of important intellectual content.

Leonardo Simas Abi Saab: Design and planning of the study.

Rúbia Tabata Rigatti: Drafting and editing of the manuscript or critical review of important intellectual content.

Oscar Cardoso Dimatos: Design and planning of the study.

Athos Paulo Santos Martini: Drafting and editing of the manuscript or critical review of important intellectual content.

## Conflicts of interest

None declared.
